# Effect of Adjuvant Chemotherapy on Survival of Patients With 8th Edition Stage IB Non-Small Cell Lung Cancer

**DOI:** 10.3389/fonc.2021.784289

**Published:** 2022-01-27

**Authors:** Yangyang Xu, Bing Wan, Suhua Zhu, Tianli Zhang, Jingyuan Xie, Hongbing Liu, Ping Zhan, Tangfeng Lv, Yong Song

**Affiliations:** ^1^ Department of Respiratory and Critical Care Medicine, Jinling Hospital, Nanjing Medical University, Nanjing, China; ^2^ Department of Respiratory and Critical Care Medicine, The Affiliated Jiangning Hospital of Nanjing Medical University, Nanjing, China; ^3^ Department of Respiratory and Critical Care Medicine, Jinling Hospital, Nanjing University School of Medicine, Nanjing, China; ^4^ Department of Respiratory and Critical Care Medicine, Jinling Hospital, Medical School of Southeast University, Nanjing, China

**Keywords:** chemotherapy, stage IB, non-small cell lung cancer, nomogram, SEER

## Abstract

**Background:**

The efficacy of adjuvant chemotherapy in patients with 8th edition stage IB (tumor size ≤4 cm) non-small cell lung cancer (NSCLC) remains unclear.

**Methods:**

We identified 9757 eligible patients (non-chemotherapy group: n=8303; chemotherapy group: n=1454) between 2004 and 2016 from the Surveillance, Epidemiology and End Results (SEER) database. Log-rank test was used to compare overall survival (OS) between the chemotherapy and non-chemotherapy groups. Cox regression model was applied to investigate the independent prognosis factors of all surgically treated stage IB patients, and then the nomogram was constructed. Propensity score matching (PSM) was performed to reduce the confounding bias, and subgroup analyses of the matched cohort were also performed. Finally, we reviewed 184 patients with stage IB NSCLC from July 2008 to December 2016 in Jinling Hospital as a validation cohort, and compared disease-free survival (DFS) and OS between the two groups.

**Results:**

In the SEER database cohort, adjuvant chemotherapy was associated with improved OS in both unmatched and matched (1417 pairs) cohorts (all P <0.05). The survival benefit (both OS and DFS) was confirmed in the validation cohort (P <0.05). Multivariate analysis showed age, race, sex, marital status, histology, tumor location, tumor size, differentiation, surgical method, lymph nodes (LNs) examined, radiotherapy and chemotherapy were prognostic factors for resected stage IB NSCLC (all P <0.05). The concordance index and calibration curves demonstrated good prediction effect. Subgroup analyses showed patients with the following characteristics benefited from chemotherapy: old age, poor differentiation to undifferentiation, 0-15 LNs examined, visceral pleural invasion (VPI), lobectomy and no radiotherapy (all P <0.05).

**Conclusions:**

Adjuvant chemotherapy is associated with improved survival in 8th edition stage IB NSCLC patients, especially in those with old age, poorly differentiated to undifferentiated tumors, 0-15 LNs examined, VPI, lobotomy and no radiotherapy. Further prospective trials are needed to confirm these conclusions. Besides, the nomogram provides relatively accurate prediction for the prognosis of resected stage IB NSCLC patients.

## Introduction

Surgical resection is the gold standard for the treatment of patients with early stage non-small cell lung cancer (NSCLC), providing patients with the greatest long-term survival opportunity (1). However, recurrence and death still occur in 30%-75% of patients even after complete resection. The 5-year survival of surgically resected NSCLC patients were reported to be 73%-90% and 56%-65% in pathologic stage I and II, respectively, and only 41% in stage IIIa (2–4). Postoperative recurrence of NSCLC is mostly systemic (about 70%), which suggests that most patients have micrometastases during surgical resection (5, 6). Therefore, many trials have been conducted worldwide to explore whether active adjuvant chemotherapy can improve the survival of early NSCLC.

Previous studies have shown that adjuvant chemotherapy has become the standard of routine care for patients with stage II and III NSCLC after radical surgery, with an improvement in 5-year survival of approximately 5% (7–9). However, whether adjuvant chemotherapy is required after surgical resection of stage IB NSCLC remains controversial. Several studies have suggested that adjuvant chemotherapy was not associated with improved survival in patients with stage IB NSCLC (based on the 6th or 7th edition of the TNM classification) (7, 10, 11). While exploratory analyses of CALGB 9633 and JBR-10 found that stage IB NSCLC patients with tumors larger than 4 cm in diameter tended to have survival benefits (6th edition) (11, 12). These studies provide evidence for adjuvant chemotherapy in stage IB NSCLC patients with tumor size ≥4 cm (6th or 7th edition). In addition, a retrospective study found patients with tumor size ≥3.2 cm may also benefit from platinum-based adjuvant chemotherapy (7th edition) (13). Another study showed that patients with completely resected T2N0M0 NSCLC (7th edition) benefited from adjuvant chemotherapy regardless of tumor size (including patients with tumor size less than 4 cm) (14). Li et al. even found that stage IB NSCLC patients with tumors <4 cm in diameter had significantly better survival than patients with tumors ≥4 cm in diameter (7th edition) (15). Therefore, there are still different views as to whether patients with tumors <4 cm in diameter can benefit from adjuvant chemotherapy. Currently, the National Comprehensive Cancer Network (NCCN) guideline recommends postoperative adjuvant chemotherapy for stage IB patients with high risk factors, such as poorly differentiated tumors, unknown lymph node status, visceral pleural invasion (VPI) and so on (16).

With the release of the eighth edition of the TNM classification, node-negative tumors between 4 and 5 cm (T2b) in diameter are reclassified as stage IIA (4). It is necessary to re-evaluate the role of adjuvant chemotherapy in stage IB disease according to the new staging system. Our study aims to explore whether patients with the latest 8th edition of stage IB NSCLC (tumor size ≤4 cm) could benefit from adjuvant chemotherapy. In addition, we attempt to establish a nomogram to predict survival outcomes in this population for better clinical decision making.

## Patients and Methods

### Study Design and Patients Source

We used SEER*Stat software (version 8.3.6) to select patients diagnosed with NSCLC from the National Cancer Institute SEER database between January 2004 and December 2016. The inclusion criteria for all patients were as follows: (I) All patients underwent surgical resection; (II) Patients with tumor size of 3-4 cm, or tumor diameter ≤3 cm but classified as T2a due to VPI were included; (III) Patients had no lymph node (LN) metastasis or distant metastasis; (IV) NSCLC was the first primary tumor; (V) All patients were histologically diagnosed as NSCLC; (VI) Complete follow-up data were obtained. The exclusion criteria were as follows: (I) Patients aged <18 years; (II) Patients underwent only local destruction, laser ablation or cryotherapy; (III) Preoperative radiotherapy was performed; (IV) Clinicopathological features (including sex, age, race, marital status, histology, location, laterality, differentiation, tumor size, VPI status, surgical method, number of LNs examined, chemotherapy and radiotherapy status) were unknown; (V) Patients had TX or T3-T4 tumors; (VI) All deaths within 6 months or follow-up time less than 6 months were excluded to rule out postoperative complications and postoperative frailty.

The included patients were divided into chemotherapy group and non-chemotherapy group according to whether they received chemotherapy or not. The following information was obtained for each patient as required by the study: basic patient information (sex, age, race and marital status), clinicopathological features of the tumor (histology, location, laterality, differentiation, tumor size and VPI status), treatment information (surgical method, number of LNs examined, chemotherapy and radiotherapy status) and survival information (survival time, follow-up outcome). Overall survival (OS) was used as evaluation index to assess the prognosis of the patients. OS was observed starting from the medical diagnosis time and ending in death of any cause.

To validate the results of the SEER database cohort, we reviewed patients with stage IB (pT2aN0M0) NSCLC who underwent surgical treatment from July 2008 to December 2016 in Jinling Hospital, Nanjing, China as a validation cohort. The inclusion criteria were the same as before. Patients with postoperative complications or postoperative frailty were excluded. All patients were followed up by telephone from the first day of diagnosis to December 19, 2019. The validation cohort was mainly to assess OS of the two groups. In addition, we reviewed patients with complete follow-up records in our hospital (including blood tests or imaging data such as chest and abdominal CT, brain magnetic resonance imaging, whole-body bone scan or positron emission tomography) and compared their disease-free survival (DFS, defined as the interval between diagnosis and tumor recurrence or death).

### Statistical Analysis

All statistical analyses were performed using R 3.6.1 statistical software. Clinicopathologic factors between chemotherapy group and non-chemotherapy group were compared *via* chi-square test. Kaplan-Meier method was used to analyze the OS and DFS, and log-rank test was used to compare survival between the two groups. The Cox regression model was applied to evaluate prognostic factors of the patients. Variables with P <0.1 in univariate analysis were included in multivariate analysis. Based on the results of multivariate analysis, the nomogram for all patients with stage IB NSCLC was constructed using the rms package. The concordance index (C-index) and calibration curves were used to measure the performance of the nomograms. Bootstraps of 1000 resamples were used for analysis.

In addition, we performed propensity score matching (PSM) analysis using the Matchlt package to make the baseline data comparable between the two groups (17). The matching variables included sex, race, age, marital status, histology, tumor location, tumor size, differentiation, VPI status, surgical methods, number of LNs examined and radiotherapy status. The caliper width is set to 0.05 standard deviation. According to the method of Strauss et al, we performed subgroup analyses defined by patient characteristics using the multivariate Cox regression model to compare OS in patients receiving chemotherapy with those undergoing surgery alone (11). P <0.05 was considered statistically significant.

## Results

### Baseline Characteristics of Patients in the SEER Database Cohort

**Figure 1:**
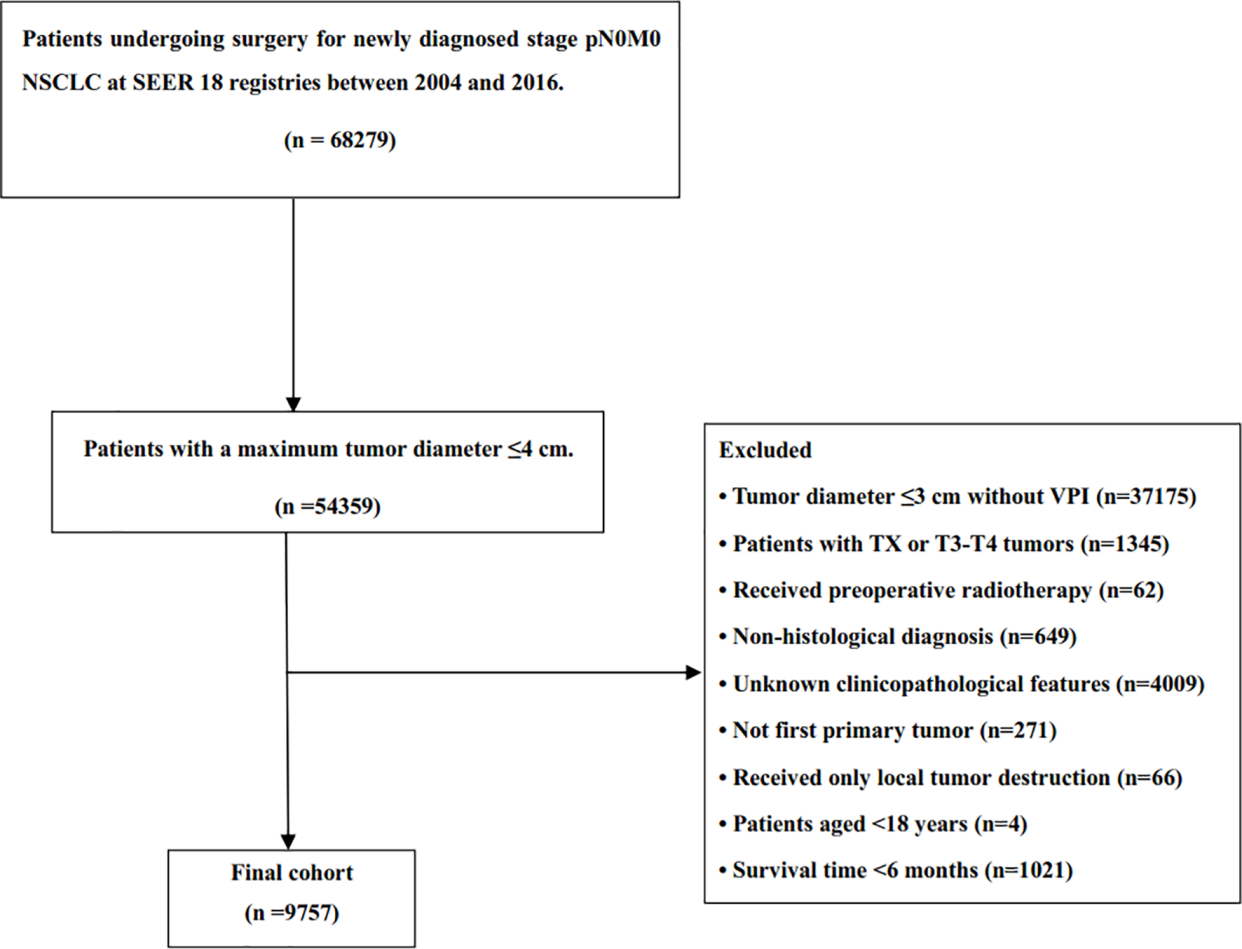
Consort diagram of patient selection from SEER database.

Of the 9757 patients identified from the SEER database, 8303 patients (85.1%) received surgery alone, and 1454 patients (14.9%) received adjuvant chemotherapy (**Figure 1**). **Table 1** shows the baseline characteristics of patients in both groups. Patients who received chemotherapy were younger (P <0.001), more likely to be married (P <0.001) and diagnosed with adenocarcinoma (P <0.001) compared with those who underwent surgery alone. Meanwhile, patients with worse degree of differentiation (P <0.001) and larger tumors (P <0.001) were more likely to receive chemotherapy. After PSM analysis, 2834 patients were successfully matched. Baseline characteristics between the two groups were well balanced (**Table 1**).

**Table 1:** Baseline characteristics of stage IB NSCLC patients in the SEER database cohort.

Characteristic	No. of patients before PSM (%)			No. of patients after PSM (%)		
	No chemotherapy group (n = 8303)	Chemotherapy group (n = 1454)	P-value	No chemotherapy group (n = 1417)	Chemotherapy group (n = 1417)	P-value
Age, years			<0.001			0.467
≤55	773 (9)	277 (19)		229 (16)	256 (18)	
56-65	2049 (25)	501 (34)		482 (34)	491 (35)	
66-75	3231 (39)	548 (38)		569 (40)	542 (38)	
≥76	2250 (27)	128 (9)		137 (10)	128 (9)	
Race			0.451			0.748
White	6943 (84)	1214 (83)		1184 (84)	1182 (83)	
Black	677 (8)	130 (9)		134 (9)	127 (9)	
Other	683 (8)	110 (8)		99 (7)	108 (8)	
Marital status			<0.001			0.059
Single	911 (11)	177 (12)		213 (15)	170 (12)	
Married	4767 (57)	913 (63)		829 (59)	890 (63)	
Widowed	1512 (18)	161 (11)		168 (12)	158 (11)	
Other	1113 (13)	203 (14)		207 (15)	199 (14)	
Gender			0.942			0.851
Male	3894 (47)	684 (47)		673 (47)	667 (47)	
Female	4409 (53)	770 (53)		744 (53)	750 (53)	
Histology			<0.001			0.493
AC	5382 (65)	999 (69)		938 (66)	971 (69)	
SCC	2184 (26)	307 (21)		342 (24)	301 (21)	
LCC	125 (2)	38 (3)		32 (2)	36 (3)	
ASC	242 (3)	42 (3)		38 (3)	42 (3)	
NET	280 (3)	38 (3)		43 (3)	38 (3)	
Other	90 (1)	30 (2)		24 (2)	29 (2)	
Tumor size, mm			<0.001			0.893
≤20	1858 (22)	274 (19)		279 (20)	271 (19)	
21-30	1879 (23)	316 (22)		311 (22)	307 (22)	
31-35	2864 (34)	459 (32)		432 (30)	451 (32)	
36-40	1702 (20)	405 (28)		395 (28)	388 (27)	
Tumor location			0.196			0.65
Upper lobe	5040 (61)	849 (58)		811 (57)	827 (58)	
Middle lobe	492 (6)	97 (7)		87 (6)	95 (7)	
Lower lobe	2657 (32)	481 (33)		496 (35)	468 (33)	
Other	114 (1)	27 (2)		23 (2)	27 (2)	
Lateral origin			0.152			1
Left	3413 (41)	568 (39)		557 (39)	558 (39)	
Right	4890 (59)	886 (61)		860 (61)	859 (61)	
Differentiation			<0.001			0.938
Well	1282 (15)	150 (10)		143 (10)	148 (10)	
Moderate	4107 (49)	645 (44)		639 (45)	632 (45)	
Poor	2763 (33)	610 (42)		593 (42)	590 (42)	
Undifferentiated	151 (2)	49 (3)		42 (3)	47 (3)	
Surgery			0.054			0.8
Sublobectomy	1390 (17)	207 (14)		213 (15)	204 (14)	
Lobectomy	6802 (82)	1225 (84)		1179 (83)	1191 (84)	
Pneumonectomy	111 (1)	22 (2)		25 (2)	22 (2)	
LNs examined, no.			0.001			0.737
0-7	4472 (54)	857 (59)		848 (60)	830 (59)	
8-15	2667 (32)	404 (28)		380 (27)	398 (28)	
≥16	1164 (14)	193 (13)		189 (13)	189 (13)	
VPI			0.469			0.421
No	3474 (42)	593 (41)		559 (39)	581 (41)	
Yes	4829 (58)	861 (59)		858 (61)	836 (59)	
Radiotherapy			<0.001			0.358
No	8067 (97)	1298 (89)		1282 (90)	1297 (92)	
Yes	236 (3)	156 (11)		135 (10)	120 (8)	

PSM, Propensity score matching; AC, Adenocarcinoma; SCC, Squamous cell carcinoma; LCC, Large cell carcinoma; ASC, Adenosquamous carcinoma; NET, Neuroendocrine tumor; LNs, Lymph nodes; No, Number; VPI, Visceral pleural invasion.

### Survival Analysis of the SEER Database Cohort Before and After PSM

**Figure 2:**
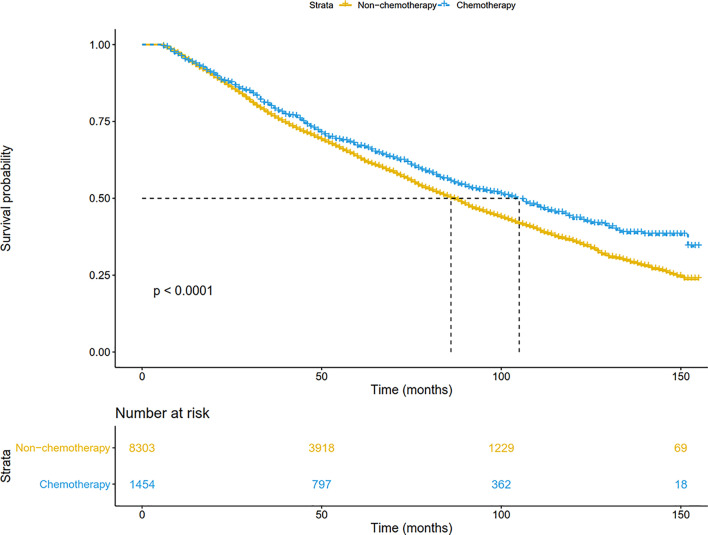
Overall survival comparison by treatment arm for stage IB NSCLC patients in the SEER database cohort.

For the entire SEER database cohort, the median follow-up period from the date of diagnosis was 48 (range, 6-155) months. Five-year survival rate for all resected stage IB NSCLC patients was 63.8%. Compared with the non-chemotherapy group, patients in the chemotherapy group had a significant survival advantage (P <0.001) (**Figure 2**). The median survival was 86 months for patients treated with surgery alone, and 105 months for those receiving chemotherapy. The 5-year survival in the chemotherapy arm (66.8%) was 3.6% higher than that in the non-chemotherapy arm (63.2%).

**Figure 3:**
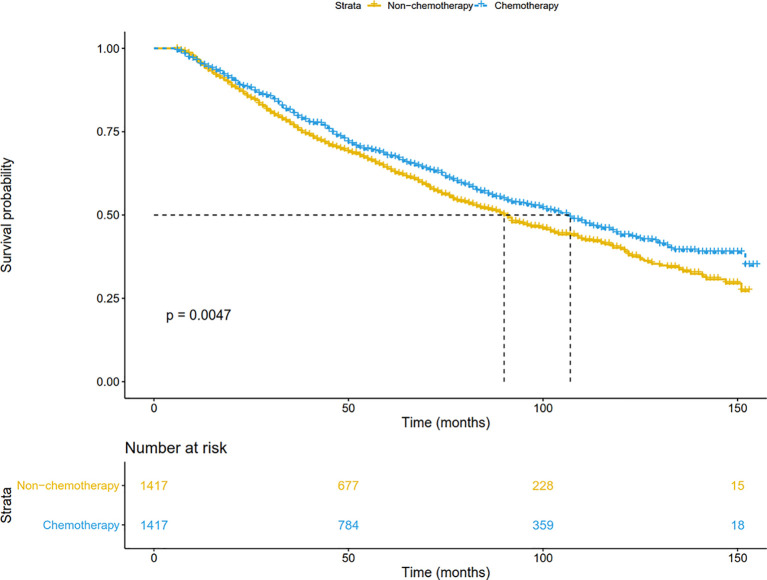
Overall survival comparison by treatment arm for stage IB NSCLC patients in the SEER database cohort after PSM. PSM, Propensity score matching.

In addition, patients were matched at 1:1 by the PSM to reduce the confounding bias. As shown in **Figure 3**, chemotherapy significantly prolonged overall survival (P=0.005). The median OS was 90 months in the non-chemotherapy group, and 107 months in the chemotherapy group. The 5-year survival in the chemotherapy arm (67.5%) was also higher than that in the non-chemotherapy arm (63.9%). After adjusting for other prognostic factors, patients receiving chemotherapy still had a more significant survival advantage than those receiving surgery alone (HR: 0.87, 95%CI: 0.78-0.98; P = 0.020).

### Prognostic Nomogram for OS in Patients With Resected Stage IB NSCLC

The factors related to long-term survival of stage IB NSCLC patients were shown in **Table 2**. The results indicated that chemotherapy was an independent prognostic factor (HR: 0.89, 95%CI: 0.81-0.97; P=0.009). In addition, patients with the following characteristics have a poor prognosis: older age (P < 0.001), diagnosis of squamous cell carcinoma (P <0.001), larger tumor size (36-40 mm: P <0.001), poorer differentiation (P <0.001), receiving sublobectomy (P <0.001), pneumonectomy (P=0.025) and radiotherapy (P <0.001). Other races (Indian, Asian, etc.) (P=0.006), married population (P=0.004), female (P <0.001) and patients with more LNs examined (P <0.001) had a better prognosis.

**Table 2:** Univariate and multivariate analyses of overall survival in all resected stage IB NSCLC patients.

Characteristic	Univariable analysis		Multivariate analysis	
	HR (95% CI)	P-value	HR (95% CI)	P-value
Age, years				
≤55	Reference		Reference	
56-65	1.47 (1.29-1.68)	<0.001	1.36 (1.19-1.56)	<0.001
66-75	1.85 (1.63-2.10)	<0.001	1.70 (1.49-1.94)	<0.001
≥76	2.81 (2.47-3.19)	<0.001	2.41 (2.11-2.76)	<0.001
Race				
White	Reference		Reference	
Black	0.92 (0.82-1.03)	0.137	0.99 (0.88-1.11)	0.836
Other	0.76 (0.68-0.87)	<0.001	0.84 (0.74-0.95)	0.006
Marital status				
Single	Reference		Reference	
Married	0.99 (0.89-1.10)	0.819	0.85 (0.77-0.95)	0.004
Widowed	1.35 (1.20-1.52)	<0.001	1.08 (0.95-1.22)	0.255
Other	1.06 (0.93-1.20)	0.392	1.00 (0.88-1.14)	0.950
Gender				
Male	Reference		Reference	
Female	0.77 (0.73-0.82)	<0.001	0.75 (0.70-0.80)	<0.001
Histology				
AC	Reference		Reference	
SCC	1.43 (1.34-1.53)	<0.001	1.17 (1.09-1.26)	<0.001
LCC	1.37 (1.12-1.68)	0.002	1.13 (0.89-1.44)	0.308
ASC	1.45 (1.23-1.71)	<0.001	1.19 (1.00-1.40)	0.048
NET	0.69 (0.56-0.86)	0.001	0.82 (0.66-1.01)	0.063
Other	1.23 (0.94-1.62)	0.131	1.07 (0.81-1.42)	0.642
Tumor size, mm				
≤20	Reference		Reference	
21-30	1.19 (1.08-1.30)	<0.001	1.14 (1.04-1.25)	0.007
31-35	1.09 (1.00-1.19)	0.039	1.10 (1.00-1.20)	0.041
36-40	1.20 (1.10-1.32)	<0.001	1.22 (1.11-1.34)	<0.001
Tumor location				
Upper lobe	Reference		Reference	
Middle lobe	1.03 (0.90-1.18)	0.655	1.10 (0.97-1.26)	0.149
Lower lobe	1.09 (1.02-1.16)	0.015	1.07 (1.00-1.14)	0.045
Other	0.96 (0.74-1.25)	0.766	0.97 (0.74-1.26)	0.801
Lateral origin				
Left	Reference			
Right	1.00 (0.94-1.07)	0.969		
Differentiation				
Well	Reference		Reference	
Moderate	1.63 (1.47-1.81)	<0.001	1.54 (1.38-1.71)	<0.001
Poor	1.88 (1.69-2.09)	<0.001	1.69 (1.51-1.89)	<0.001
Undifferentiated	2.00 (1.62-2.46)	<0.001	1.79 (1.40-2.29)	<0.001
Surgery				
Lobectomy	Reference		Reference	
Pneumonectomy	1.20 (0.94-1.53)	0.134	1.33 (1.04-1.71)	0.025
Sublobectomy	1.73 (1.60-1.86)	<0.001	1.44 (1.33-1.56)	<0.001
LNs examined, no.				
0-7	Reference		Reference	
8-15	0.76 (0.71-0.81)	<0.001	0.82 (0.76-0.88)	<0.001
≥16	0.69 (0.62-0.77)	<0.001	0.72 (0.65-0.80)	<0.001
VPI				
No	Reference			
Yes	1.03 (0.97-1.09)	0.384		
Radiotherapy				
No	Reference		Reference	
Yes	1.71 (1.50-1.94)	<0.001	1.45 (1.27-1.66)	<0.001
Chemotherapy				
No	Reference		Reference	
Yes	0.82 (0.75-0.90)	<0.001	0.89 (0.81-0.97)	0.009

AC, Adenocarcinoma; SCC, Squamous cell carcinoma; LCC, Large cell carcinoma; ASC, Adenosquamous carcinoma; NET, Neuroendocrine tumor; LNs, Lymph nodes; VPI, Visceral pleural invasion; HR, Hazard ratio; CI, Confidence interval.

**Figure 4:**
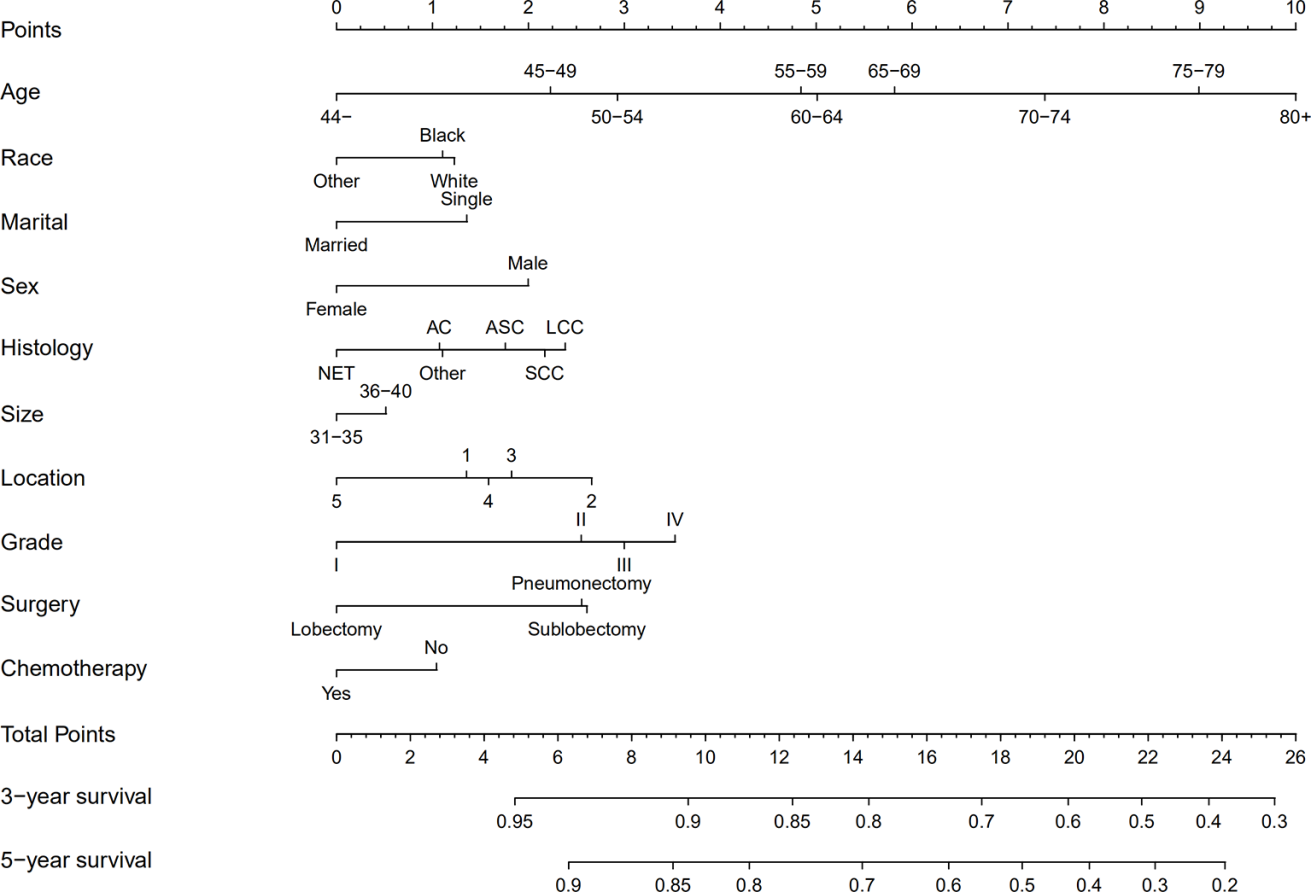
Nomograms for predicting the 3- and 5-year OS rates of patients with stage IB NSCLC undergoing surgery. AC, Adenocarcinoma; SCC, Squamous cell carcinoma; LCC, Large cell carcinoma; ASC, Adenosquamous carcinoma; NET, Neuroendocrine tumor; LNs, Lymph nodes; OS, Overall survival; Grade I, Well differentiation; Grade II, Moderate differentiation; Grade III, Poor differentiation; Grade IV, Undifferentiation.

**Figure 5:**
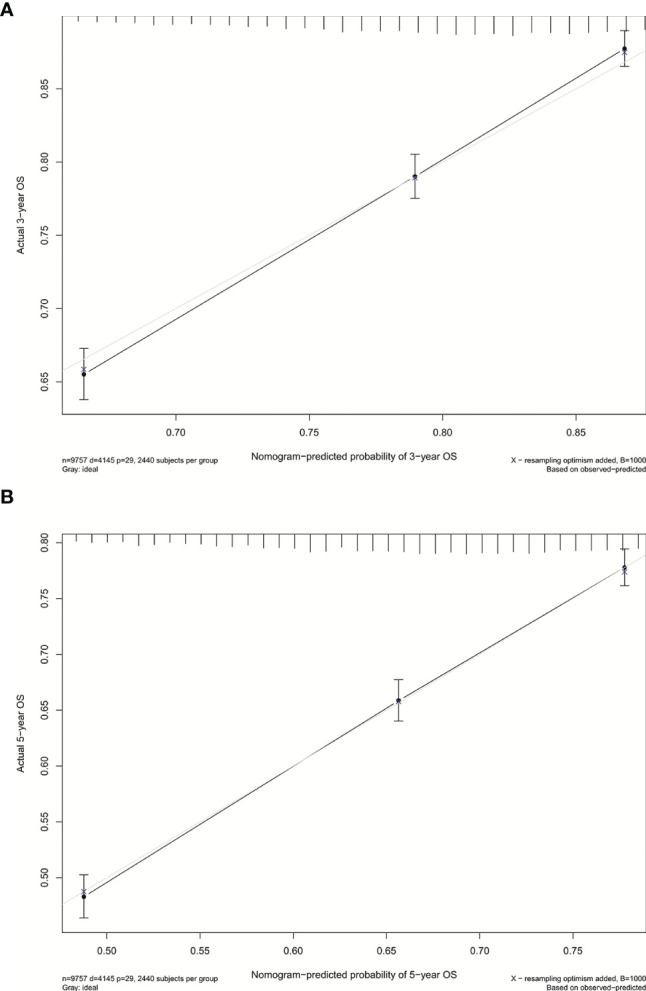
**(A)** Calibration curve for predicting 3-year OS rates of patients with stage IB NSCLC undergoing surgery. **(B)** Calibration curve for predicting 5-year OS rates of patients with stage IB NSCLC undergoing surgery.

We established the nomogram for all surgically resected stage IB NSCLC patients to predict their survival (**Figure 4**). The 3-year or 5-year OS can be estimated by adding the score assigned to each characteristic. The C-index was 0.640 for the nomogram of all patients. In addition, the calibration curves of the nomogram (**Figure 5**) showed their consistency.

### Survival Benefit of Chemotherapy According to Patient Characteristics in the Matched Cohort

**Figure 6:**
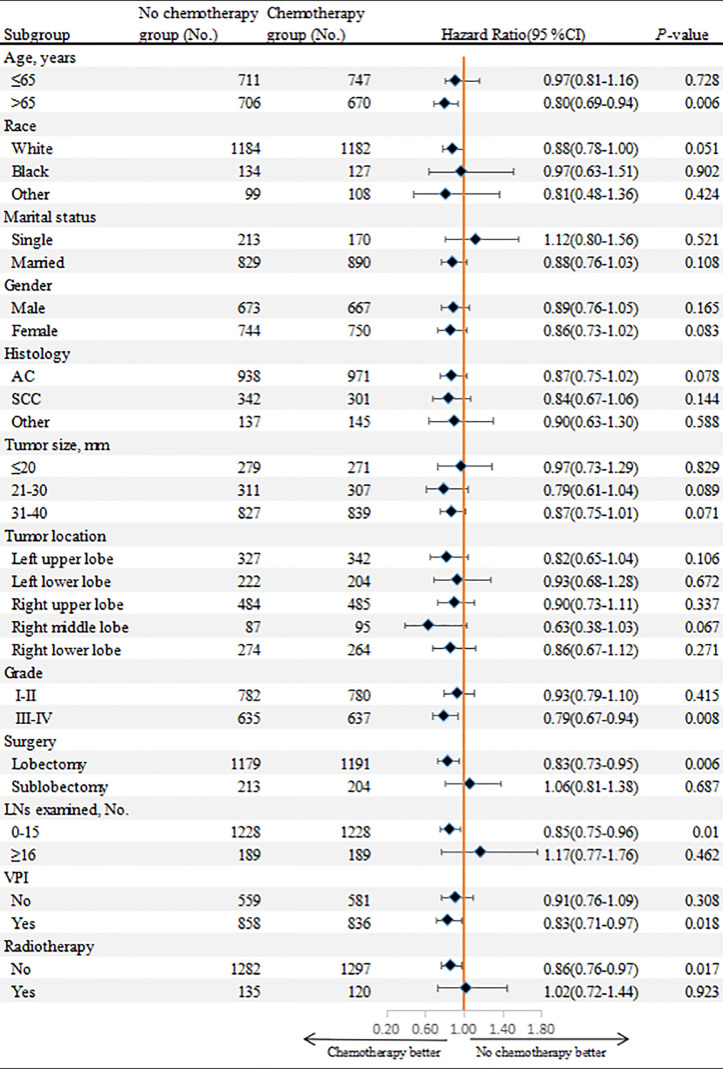
Overall survival comparisons between chemotherapy and non-chemotherapy groups according to patient characteristics in the matched cohort.

To further clarify the role of adjuvant chemotherapy in patients with stage IB NSCLC, we performed subgroup analyses in the matched cohort (**Figure 6**). The survival benefit of chemotherapy was superior to that of surgery alone for patients with old age (>65 years, P=0.006), poor-differentiated to undifferentiated NSCLC (P=0.008), VPI (P=0.018), 0-15 LNs examined (P=0.01) and lobectomy performed (P=0.006). In addition, for patients without radiotherapy (P=0.017), adjuvant chemotherapy had a survival advantage over surgery alone.

### Baseline Characteristics and Survival Analysis of Validation Cohort

From the single medical center, 184 patients were finally included. Of these, 104 (56.5%) cases were treated with surgery alone, and 80 (43.5%) cases received adjuvant chemotherapy. Although we did not limit the treatment regimen, none of the patients received radiotherapy. As shown in Table 3, patients who received chemotherapy were more likely to be male (75% vs. 60%, P=0.042), poor-differentiated to undifferentiated (39% vs. 23%, P=0.047), have larger tumor size (88% vs. 69%, P=0.006) and more LNs examined (45% vs. 30%, P=0.003) compared with those who received surgery alone. After PSM analysis, 104 patients were successfully matched.

**Figure 7:**
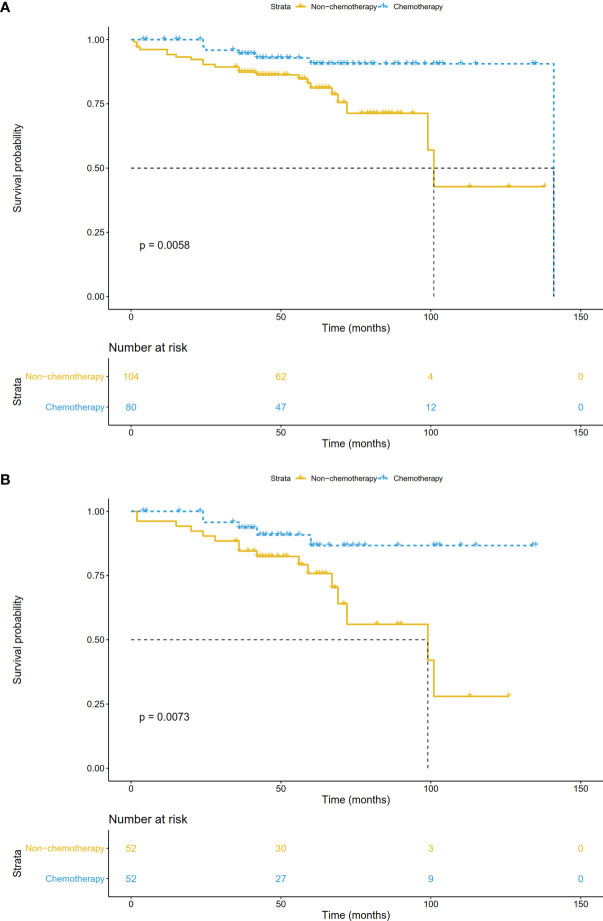
Overall survival comparisons by treatment arm for stage IB NSCLC patients in the validation cohort **(A)** before PSM and **(B)** after PSM.

**Figure 8:**
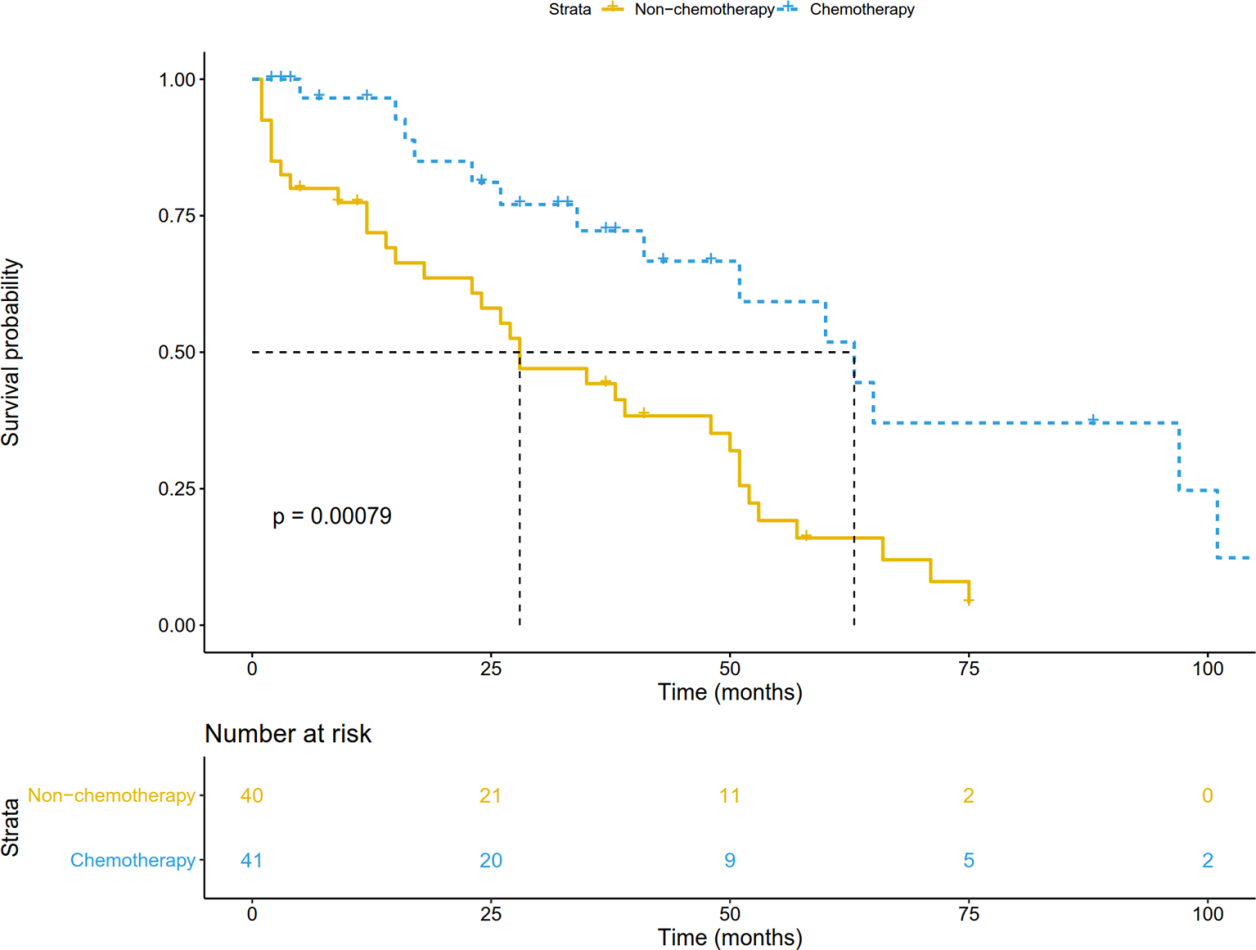
Disease-free survival comparison by treatment arm for stage IB NSCLC patients in the validation cohort.

In the validation cohort, the median follow-up was 57.5 (range, 1-141) months. The survival curve showed that adjuvant chemotherapy was associated with improved OS (P=0.006) (**Figure 7A**). After adjusting for other prognostic factors, patients receiving adjuvant chemotherapy still had a significant survival advantage (HR: 0.23, 95%CI 0.09-0.58; P=0.002). In the matched cohort, chemotherapy also significantly prolonged OS (P=0.007) (**Figure 7B**). In addition, we obtained DFS information in 81 patients, and their clinical features were shown in **Supplementary Table 1**. There was no significant difference between the two groups except for age. Survival analysis showed that patients receiving adjuvant chemotherapy had longer DFS (P <0.001) (**Figure 8**).

### Sensitivity Analysis

In the SEER database, information on whether or not the patient received chemotherapy was reported as “no/unknown” and “yes”. A previous study compared the SEER database with the SEER-Medicare database and found the sensitivity of chemotherapy variables in lung cancer was 80.1% (18). In other words, about 20% of the patients in the non-chemotherapy group in this study were also likely to have received chemotherapy.

To assess the consistency of the study results, we performed the following sensitivity analysis: 20% of patients in the non-chemotherapy group of the SEER database cohort were reclassified into the chemotherapy group for survival analysis, which was repeated 1000 times. Finally, 86.2% of the simulation results showed that the overall survival of patients receiving chemotherapy was significantly higher than that of patients without chemotherapy. This indicates that the bias in the results of OS caused by the misclassification of whether or not to receive chemotherapy is small.

## Discussion

The ADAURA study showed that adjuvant targeted therapy, which is only applied to driver mutation-positive NSCLC, can improve disease-free survival in NSCLC patients, while the OS benefit remains unclear (19). In addition, adjuvant immunotherapy for NSCLC is still in the exploratory stage, and there are no mature research results. Therefore, adjuvant chemotherapy still plays an important role in the treatment of early stage lung cancer. So far, adjuvant chemotherapy is recommended for patients with resectable stage II, IIIA or IB large tumors (>4 cm in 7th edition). However, whether patients with the latest 8th edition of stage IB NSCLC (tumor size ≤4 cm) can benefit from adjuvant chemotherapy remains unclear.

There have been only a few studies specifically focused on the role of adjuvant chemotherapy in patients with lymph node-negative tumors less than 4 cm in diameter, and most of them are exploratory analyses. Wang et al. retrospectively analyzed 402 patients with 8th edition stage IB NSCLC. They found that patients receiving adjuvant chemotherapy (137 cases) had worse OS than those receiving observation alone (265 cases) (20). One retrospective study involving 211 patients also showed that adjuvant chemotherapy did not improve survival in patients with 8th edition stage IB NSCLC. However, only 20 people in the study received adjuvant chemotherapy, so the conclusions should be taken with caution (21). Another study included a total of 119 7th edition stage IB patients who underwent complete surgical resection. Subgroup analysis suggested that adjuvant chemotherapy was associated with better survival in patients with tumors ≥ 3.2 cm (13). In addition, two studies based on the National Cancer Data Base (NCDB) found patients with node-negative tumors <4 cm in diameter could also benefit from adjuvant chemotherapy (14, 22).

In our study including 9757 resectable 8th edition stage IB NSCLC patients, 8303 underwent surgery alone and 1454 received adjuvant chemotherapy. Survival analysis of both unmatched and matched cohorts showed adjuvant chemotherapy significantly improved OS. In addition, we reviewed 184 stage IB NSCLC patients who underwent surgical treatment in Jinling Hospital as a validation cohort. Survival analysis also showed that patients receiving adjuvant chemotherapy had a better prognosis. Further analysis found that adjuvant chemotherapy was associated with better DFS, suggesting that adjuvant chemotherapy can improve OS by preventing tumor recurrence. In the future, it may be necessary for physicians to consider adjuvant chemotherapy in the treatment of patients with stage IB NSCLC.

Nomogram is a visual statistical prediction model which can generate the prediction value of clinical events by integrating biological and clinical variables, and is widely used to predict the prognosis of cancer patients (23). The nomograms of postoperative NSCLC patients included the prediction of recurrence-free survival in stage IA NSCLC, the recurrence risk of stage I lung adenocarcinoma, the prediction of OS in early stage (stage I-II) NSCLC patients and so on, but there is no specific nomogram for resectable stage IB NSCLC (24–26). Therefore, we sought to establish a nomogram to provide a convenient and reliable model for predicting survival of patients with resectable stage IB NSCLC. Through Cox regression analysis, we identified age, race, sex, marital status, histology, tumor location, tumor size, differentiation, surgical method, LNs examined, radiotherapy and chemotherapy as independent prognostic factors for resected stage IB patients, which were highly consistent with previous studies on risk factors for NSCLC (27–29). Notably, VPI status was not found to be an independent prognostic factor for 8th edition stage IB patients in our study. This may be because the effect of VPI on the survival of NSCLC is related to tumor size and is affected by the follow-up treatment (30–32). David et al. retrospectively analyzed 1166 patients with pathologic N0M0 NSCLC and found that VPI was not significantly associated with survival for tumors <5 cm, whereas VPI was a poor prognostic factor for disease-free survival for patients with stage T2b and T3 tumors (30). Similarly, some other studies have also found no significant effect of VPI on OS of stage I NSCLC, which is consistent with the conclusion of this study (31, 32). Then based on the results of the above regression analysis, we constructed the nomogram. The C-index of 0.640 indicated that this nomogram could effectively predict the 3- and 5-year survival rate. To our knowledge, this is currently the first nomogram for 8th edition resected stage IB patients, which has important reference value.

In the subgroup analysis, we found that although the prognosis of elderly NSCLC patients receiving adjuvant chemotherapy (>65 years old) was poor, adjuvant chemotherapy still improved the prognosis of these patients compared to surgery alone. The JBR.10 study showed that the survival benefit of adjuvant chemotherapy could be observed even in patients ≥65 years old (HR: 0.61, 95%CI: 0.38-0.98; P=0.04) (33). Another study, involving 7593 patients with early-stage NSCLC, showed that patients aged ≥70 years also benefited from adjuvant chemotherapy (34). Therefore, adjuvant chemotherapy should not be rejected on the basis of age alone. For patients undergoing lobectomy, adjuvant chemotherapy was associated with better survival, while no survival benefit of adjuvant chemotherapy was observed in patients undergoing sublobectomy. This may be because most patients undergoing sublobectomy have more severe comorbidities and may not tolerate adjuvant chemotherapy (2). Chemotherapy was associated with better survival when the number of LNs examined was less than 16, whereas there was no statistical difference in OS between the two groups when the number of LNs examined was 16 or more. Previous studies have investigated the impact of the number of LNs examined on survival of resected NSCLC, and the relationship between examined LN count and adjuvant chemotherapy. They suggested that patients with inadequate LNs examined were at high risk of undetected positive LNs, which may lead to incorrect staging of the tumor and poor survival, and thus postoperative chemotherapy provided a survival advantage for these patients (35, 36). Therefore, chemotherapy was recommended in clinical practice for stage IB NSCLC with insufficient LNs examined (<16 LNs in our study). In addition, our study suggested that patients with VPI and poorly differentiated to undifferentiated tumors could also benefit from adjuvant chemotherapy. Previous studies showed that VPI was associated with increased risk of local recurrence and systemic metastasis in lung cancer, and poorly differentiated patients have higher risk of recurrence and death after surgical resection (37–39). Therefore, adjuvant chemotherapy can eliminate minimal residual lesions in these patients, reduce the risk of recurrence, and improve survival.

Our study has several limitations. First, information that may be relevant to the prognosis of patients after surgical resection was not available, including smoking status, surgical complications, lymphovascular invasion, performance status and driver mutations, etc. Second, the information about chemotherapy given in the SEER database cannot distinguish between neoadjuvant and adjuvant chemotherapy. Previous studies have shown no difference in survival between neoadjuvant and adjuvant chemotherapy for patients with resectable lung cancer (40). Moreover, neoadjuvant chemotherapy is not the standard treatment for stage IB NSCLC. Therefore, we believed that almost all patients in the SEER database cohort received adjuvant chemotherapy. In addition, we reviewed patients receiving postoperative adjuvant chemotherapy in Jinling Hospital as the validation cohort. Third, information on chemotherapy is reported as “no/unknown” and “yes” in the SEER database. We performed sensitivity analysis based on the previous study comparing the SEER database with the SEER-Medicare database, and found that the results were reliable. Meanwhile, the survival result was confirmed in the validation cohort. Finally, our study is retrospective, and large prospective controlled clinical studies are necessary for further validation.

In conclusion, our study suggests that 8th edition stage IB NSCLC patients (tumor size ≤4 cm) could benefit from adjuvant chemotherapy, especially for patients with old age, poorly differentiated to undifferentiated tumors, 0-15 LNs examined, VPI, lobotomy and no radiotherapy. In the future, these conclusions need to be further confirmed in prospective trials.

## Data Availability Statement

The original contributions presented in the study are included in the article/**Supplementary Material**, further inquiries can be directed to the corresponding authors.

## Ethics Statement 

The studies involving human participants were reviewed and approved by Ethics Committee of Nanjing Jinling Hospital (No. 2019NZGKJ-132). Written informed consent for participation was not required for this study in accordance with the national legislation and the institutional requirements.

## Author Contributions

Conception and design: YX, BW, and PZ. Administrative support: PZ, TL, and YS. Provision of study materials or patients: HL, PZ, TL, and YS. Collection and assembly of data: YX, SZ, TZ, and JX. Data analysis and interpretation: YX, BW, and SZ. Manuscript writing: All authors. All authors contributed to the article and approved the submitted version.

## Funding

This work was supported by grants from the National Natural Science Foundation of China (grant number 81401903, 81572937 and 81572273); the16^th^ batch “Summit of the Six Top Talents” Program of Jiangsu Province (grant number WSN-154); China Postdoctoral Science Foundation 12^th^ batch Special fund (postdoctoral number 45786); China Postdoctoral Science Foundation 64^th^ batch (postdoctoral number 45786); Jiangsu Provincial Postdoctoral Science Foundation (grant number 2018K049A); the Natural Science Foundation of Jiangsu province (grant number BK20180139, BK20161386); Jiangsu Provincial Medical Youth Talent (grant number QNRC2016125); the Nanjing Medical Science and Technology Development Project (No. ZKX17044); and the Jiangsu Provincial Key Research and Development Program (No. BE2016721). 

## Conflict of Interest

The authors declare that the research was conducted in the absence of any commercial or financial relationships that could be construed as a potential conflict of interest.

## Publisher’s Note

All claims expressed in this article are solely those of the authors and do not necessarily represent those of their affiliated organizations, or those of the publisher, the editors and the reviewers. Any product that may be evaluated in this article, or claim that may be made by its manufacturer, is not guaranteed or endorsed by the publisher.
