# Supplementary material for: Effect of Adjuvant Chemotherapy on Survival of Patients With 8th Edition Stage IB Non-Small Cell Lung Cancer
Source: Front Oncol. 2022 Jan 27;11:784289. doi: 10.3389/fonc.2021.784289 (PMC8828472; doi:10.3389/fonc.2021.784289)
Supplement: Supplementary file 1 [file Table_1.docx]

Supplementary Table 1. Baseline characteristics of stage IB NSCLC patients with DFS information in the validation cohort.

| Characteristic | No chemotherapy group (n=40) | Chemotherapy group (n=41) | P-value |
| --- | --- | --- | --- |
| Age, years |  |  | 0.02 |
| < 65 | 16(40) | 28(68) |  |
| ≥ 65 | 24(60) | 13(32) |  |
| Marital status |  |  | 0.616 |
| Married | 38(95) | 40(98) |  |
| Divorced | 2(5) | 1(2) |  |
| Gender |  |  | 0.944 |
| Male | 28(70) | 30(73) |  |
| Female | 12(30) | 11(27) |  |
| Histology |  |  | 0.326 |
| AC | 29(72) | 27(66) |  |
| SCC | 8(20) | 13(32) |  |
| Other | 3(8) | 1(2) |  |
| Tumor size, mm |  |  | 0.738 |
| ≤ 30 | 9(22) | 7(17) |  |
| 31-40 | 31(78) | 34(83) |  |
| Tumor location |  |  | 0.288 |
| Upper lobe | 19(48) | 26(63) |  |
| Middle lobe | 4(10) | 5(12) |  |
| Lower lobe | 16(40) | 9(22) |  |
| Other | 1(2) | 1(2) |  |
| Lateral origin |  |  | 0.725 |
| Left | 16(40) | 19(46) |  |
| Right | 24(60) | 22(54) |  |
| Grade |  |  | 0.239 |
| I-II | 21(52) | 14(34) |  |
| III-IV | 10(25) | 13(32) |  |
| Unknown | 9(22) | 14(34) |  |
| Surgery |  |  | 0.713 |
| Sublobectomy | 1(2) | 3(7) |  |
| Lobectomy | 37(92) | 37(90) |  |
| Pneumonectomy | 2(5) | 1(2) |  |
| LNs examined, no. |  |  | 0.064 |
| 0-7 | 6(15) | 9(22) |  |
| 8-15 | 23(57) | 13(32) |  |
| ≥ 16 | 11(28) | 19(46) |  |
| VPI |  |  | 0.197 |
| No | 25(62) | 32(78) |  |
| Yes | 15(38) | 9(22) |  |

**Abbreviations:** DFS, Disease-free survival; AC, Adenocarcinoma; SCC, Squamous cell carcinoma; Grade I, Well differentiation; Grade II, Moderate differentiation; Grade III, Poor differentiation; Grade IV, Undifferentiation; LNs, Lymph nodes; No, Number; VPI, Visceral pleural invasion.
